# Frequency of Blood Eosinophilia in newly diagnosed Chronic Obstructive Pulmonary Disease patients

**DOI:** 10.12669/pjms.36.4.1624

**Published:** 2020

**Authors:** Ghulam Maqtada Khan, Faisal Faiyaz Zuberi, Syeda Bizat uz Zahra, Lubna Ghafoor

**Affiliations:** 1 Dr. Ghulam Maqtada Khan, MBBS, FCPS (Pulmonology). Trainee, Pulmonology & Chest Unit-II, Ojha Institute of Chest Diseases, Dow University of Health Sciences, Karachi, Pakistan; 2 Dr. Faisal Faiyaz Zuberi, MBBS, FCPS (Med), FCPS (Pulm), FCCP Associate Professor, Pulmonology & Chest Unit-II, Pulmonology & Chest Unit-II, Ojha Institute of Chest Diseases, Dow University of Health Sciences, Karachi, Pakistan; 3 Dr. Syeda Bizat uz Zahra, MBBS, FCPS (Pulmonology). Trainee, Pulmonology & Chest Unit-II, Ojha Institute of Chest Diseases, Dow University of Health Sciences, Karachi, Pakistan; 4 Dr. Lubna Ghafoor, MBBS, MD (Pulmonology). Trainee, Pulmonology & Chest Unit-II, Ojha Institute of Chest Diseases, Dow University of Health Sciences, Karachi, Pakistan

**Keywords:** Eosinophilia, Chronic Obstructive Pulmonary Disease, Spirometry

## Abstract

**Objective:**

To determine Blood Eosinophilia frequency in newly diagnosed Chronic Obstructive Pulmonary Disease patients.

**Methods:**

An observational cross-sectional research on newly diagnosed COPD patients with age ≥ 40 years was performed at Ojha Institute of Chest Diseases, Dow University of Health Sciences Karachi. COPD patients diagnosed in outpatient clinic or admitted in Chest Unit during six-month period from September 2018 to May 2019 were selected. Each patient underwent spirometry, and blood was tested for eosinophilia (≥ 2% eosinophils in blood), after obtaining informed consent and clinical history.

**Results:**

One hundred and fifty COPD patients were diagnosed and evaluated for *Blood Eosinophilia*, out of which 86 (57.3%) and 64 (42.7%) patients were *Males* and *Females* respectively with mean age of 63.72 ± 10.24 years. *Current Smokers* were 48 (32.0%), *Ex-Smokers* 15 (10.0%), and *Biomass Exposure* was present in 76 (50.7%) of patients. Spirometric severity of COPD was *Mild* in 11 (7.3%), *Moderate* in 68 (45.3%), *Severe* in 59 (39.3%), and *Very Severe* in 12 (8.0%) patients. *Blood Eosinophilia* was present in 59 (39.3%) patients of COPD among which majority 43 (72.9%) were having *Moderate* 24 (40.7%) and *Severe* 19 (32.2%) COPD respectively.

**Conclusion:**

Frequency of Blood Eosinophilia in newly diagnosed COPD patients was high, among which most of the COPD patients were moderate to severe.

## INTRODUCTION

Chronic Obstructive Pulmonary Disease (COPD) is among the heterogenous diseases associated with different types of clinical as well as pathophysiologic phenotypes.^1^,^2^ Global Initiative for Chronic Obstructive Lung Disease (GOLD) defined the COPD as “A common, preventable and treatable disease that is characterized by persistent respiratory symptoms and airflow limitation that is due to airway and/or alveolar abnormalities usually caused by significant exposure to noxious particles or gases”.^3^ According to World Health Organization (WHO) recent report on COPD; approximately 251 million people are suffering from COPD throughout the world, whereas 5% of total death (3.17 million) occurs due to COPD in year 2015, among which 90% were reported from developing countries.^4^ Today, COPD is considered as the 4^th^ leading cause of mortality and it will become the 3^rd^ in 2030.^5^,^6^ It is included in incurable diseases, but its progression can be slow down with reducing exposure to risk factors, and with early diagnosis, and proper management. Exposure of healthy persons to tobacco smoke is the most common COPD risk factor followed by older age, male gender as well as exposure to indoor or outdoor pollution.^7^

Early diagnosis of COPD requires the identification of non-invasive and reliable biomarkers that helps in identification of different types of COPD phenotypes resulting in appropriate and specific treatment.^8^ Increased blood eosinophils have been reported in COPD patients, playing vital role in COPD pathogenesis.^8^-^10^ Eosinophilia is diagnosed in COPD patients on presence of ≥ 2% eosinophils in blood. With recent development, role of controlling blood eosinophilia in COPD management has been increased. COPD with increased eosinophilia is also used as a biomarker for exacerbations.^11^-^13^ Although exact prevalence of eosinophilia in COPD patients is still unknown, few researchers reports the prevalence of eosinophilia in approximately 20% COPD patients. Negewo NA, et al., reports blood eosinophilia in 15.6% COPD patients,^9^ whereas Hasegawa K, et al., reports in 17% COPD patients.^14^

The rationale of this study is to document presence of blood eosinophilia in newly diagnosed cases of COPD for appropriate treatment of COPD patients, which is critical to reduce the progression of COPD and improve the outcomes. Only newly diagnosed cases of COPD were included as the eosinophil count in blood will not be affected by any treatment taken for COPD and so does its disease course. Different factors affect the response to treatment in COPD patients and knowing these may help in proper and timely management and reducing associated morbidity and mortality, blood eosinophilia is also considered among such factors. COPD patients with blood eosinophilia should be managed with inhaled corticosteroids and this will have better symptoms relief and the disease progression will be slowed also. Different evidence suggested that high eosinophil in blood is directly correlated with increased risk of exacerbations in COPD patients. Management of eosinophilic COPD with inhaled corticosteroid also reduces exacerbations. Recently, no such study has been performed in Pakistan on blood eosinophilia in newly diagnosed COPD patients. Therefore, this study was conducted to document frequency of blood eosinophilia in newly diagnosed COPD patients.

## METHODS

An observational cross-sectional research on newly diagnosed COPD patients was performed at Ojha Institute of Chest Diseases, Dow University of Health Sciences (DUHS) Karachi. COPD patients admitted in Chest Unit during the period of six months from 8^th^ September 2018 to 8^th^ May 2019 were selected by purposive sampling. COPD patients with age ranging from 40 to 80 years, were included whereas patients taking tuberculosis therapy or having Asthma, Asthma COPD overlap (ACO) and Bronchiectasis were excluded from study. The sample size was calculated through open EPI software by using the proportion of Hasegawa K, et al., 17% patients with blood eosinophilia in COPD patients,^14^ taking confidential interval 95% and margin of error 6%, sample size becomes n = 150. The synopsis of the study was approved by the REU of CPSP Ref. CPSP/REU/Pul-2016-212-372 dated Nov. 8, 2018.

COPD was confirmed in patients according to GOLD criteria such as clinical history and spirometry showing persistent airflow limitation (ratio of FEV_1_ / FVC < 0.70 pre- and post-bronchodilator). Classification of severity of COPD as per spirometry is given in Table-I. Eosinophilia is defined as presence of ≥ 2% eosinophils in blood. A person with COPD and blood eosinophils ≥ 2% was labelled Eosinophilic COPD. A person reported with smoking of more than twenty cigarettes packs in whole life or more than one cigarettes per day for one year was labeled Current Smoker; whereas smoker who quit smoking at least one year before inclusion in study was labeled as Ex-Smoker; and a person who never smoked or smoked less than one cigarette per day for less than a year was labeled as Non-Smoker. A person who was exposed to indoor wood fire for cooking or heating or chemicals for at least 6 months was labeled as having Biomass Exposure.

**Table-I:** Severity of COPD (Post-bronchodilator values).

GOLD 1	Mild	FEV_1_ ≥ 80% predicted
GOLD 2	Moderate	50% ≤ FEV_1_< 80% predicted
GOLD 3	Severe	30% ≤ FEV_1_< 50% predicted
GOLD 4	Very Severe	FEV_1_< 30% predicted

After selection of suspected COPD patients, detailed demographic and clinical history including age, gender, occupation, smoking status and biomass exposure was collected from patients. Spirometry of each patient was performed for confirmation of COPD. Spirometry was performed by trained spirometry technician having at least 03 years’ experience, whereas interpretation was done by the consultant pulmonologist having 03 years’ experience. Blood sample of each patient was collected in aseptic environment and sent to laboratory for CBC (Complete Blood Count) for confirmation of eosinophilia. Laboratory results of each patient including FEV_1_, FVC, FEV_1_/FVC, severity of COPD (Mild, Moderate, Severe, Very Severe), Eosinophil count and Eosinophilia (yes/no) were collected and further analyzed with SPSS software version 22. Descriptive statistics for age, FEV_1_/FVC ratio and eosinophil count, and frequency and percentages of gender, age, occupation, risk factor (smoking status and biomass exposure), severity of COPD and eosinophilia was calculated. Chi-square Test was applied for determining difference in frequency of eosinophilia with risk factors like gender, age, smoking status, biomass exposure and severity of COPD. P value <0.05 was taken as significant.

## RESULTS

In this study, initially data of patients was collected and evaluated for confirmation of COPD, followed by confirmation of eosinophilia in newly diagnosed patients of COPD. Table-II shows the demographic data of diagnosed COPD patients obtained during evaluation of COPD. There were 86 (57.3%) males, and 64 (42.7%) females. Most of the COPD patients were in age group of 61-70 years, 49 (32.7%) followed by 71-80 years, 37 (24.7%), 51-60 years, 35 (23.3%), and 41-50 years 29 (19.3%). Most of the COPD patients were Non-Smokers 87 (58.0%) followed by Current-Smokers 48 (32.0%), and Ex-Smokers 15 (10.0%). Roughly half of the COPD patients were exposed to Biomass 76 (50.7%).

**Table-II:** Demographic Data of COPD Patients.

Variables	Frequency (n=150)	Percentage
*Gender*		
Male	86	57.3
Female	64	42.7
*Age*		
41-50	29	19.3
51-60	35	23.3
61-70	49	32.7
71-80	37	24.7
*Smoking*		
Current-smoker	48	32.0
Ex-smoker	15	10.0
Non-smoker	87	58.0
*Biomass Exposure*		
Yes	76	50.7
No	74	49.3

The descriptive statistics of different continuous variables obtained during collection of demographic data like age with mean of 63.72 ±10.24 years, and clinical characteristics like spirometric variables (FEV1 53.4 ±15.6%, FVC 78.3 ±16.7%, and FEV1/FVC 49.7 ±12.7%), and blood eosinophils count with mean of 3.9 ±2.7% obtained during spirometry and CBC respectively are shown in Table-III.

**Table-III:** Descriptive Statistics of Continuous Variables.

Variable	Min.	Max.	Mean	Std. Deviation
Age (Years)	45	80	63.72	10.24
FEV_1_ (%)	14.0	58.0	53.4	15.6
FVC (%)	29.0	88.0	78.3	16.7
FEV_1_/FVC (%)	34.6	86.3	49.7	12.7
Eosinophil Count (%)	1.0	10.0	3.9	2.7

According to GOLD standard, newly diagnosed COPD patients were categorized into; *Mild* 11 (7.3%), Moderate 68 (45.3%), Severe 59 (39.3%), and *Very Severe* 12 (8.0%). *Eosinophilic COPD* was confirmed in 59 (39.3%) patients, and *Non-eosinophilic COPD* in 91 (60.7%) patients. In *Eosinophilic COPD*, spirometric severity was *Mild* 8 (13.6%), *Moderate* 24 (40.7%), *Severe* 19 (32.2%), and Very Severe 8 (13.6%) (Table-IV). The non-significant difference between Eosinophilic COPD and Non-eosinophilic COPD with Gender (p=0.3), Age (p=0.3), Smoking (p=0.9), and Biomass Exposure (p=0.2), whereas significant difference with the severity of COPD (p=0.01) is also shown in Table-IV.

**Table-IV:** Risk Factors with Eosinophilia.

Variables	Eosinophilic COPD (%) (≥ 2 Eosinophil)	Non-eosinophilic COPD (%) (< 2 Eosinophil)	P-value (Chi-square test)
*Gender*			
Male	31 (52.5)	55 (60.4)	0.3
Female	28 (47.5)	36 (39.4)
*Age*			
41-50	8 (13.6)	21 (23.1)	0.3
51-60	14 (23.7)	21 (23.1)	
61-70	19 (32.2)	30 (33.0)	
71-80	18 (30.5)	19 (20.9)
*Smoking*			
Current-smoker	18 (30.5)	30 (33.0)	0.9
Ex-smoker	6 (10.2)	9 (9.9)	
Non-smoker	35 (59.3)	52 (57.1)
*Biomass Exposure*			
Yes	33 (55.9)	43 (47.3)	0.2
No	26 (44.1)	48 (52.7)
*COPD Severity*			
Mild	8 (13.6)	3 (3.3)	0.01
Moderate	24 (40.7)	44 (48.4)	
Severe	19 (32.2)	40 (44.0)	
Very severe	8 (13.6)	4 (4.4)	

## DISCUSSION

COPD is complex group of disease characterized with chronic respiratory symptoms and permanent airflow limitation, directly associated with increased morbidity and mortality. Early recognition of clinical phenotypes of COPD with use of validated biomarkers such as blood eosinophilia is very important for development of specific therapeutic strategies and appropriate management of COPD.^15^,^16^

Blood eosinophils are used as a prognostic biomarker in COPD patients. Increased blood eosinophils are directly associated with worsening of COPD, increased hospital admission, and mortality. Early identification of raised blood eosinophils in COPD patients is very important not only for appropriate management but also for lowering the risk of severe exacerbations.^9^-^12^ Therefore, in this study new COPD patients were diagnosed and their blood eosinophilia frequency was determined, which is critical to reduce the progression of COPD and to improve the outcome. Current study focuses on two important findings. Firstly, identification of COPD on the basis of GOLD standard and associated risk factors, and secondly, detection of Eosinophilic COPD in these patients.

In current study 150 newly diagnosed COPD patients were evaluated, out of which 86 (57.3%) patients were males and 64 (42.7%) were females with mean age of 63.72 ±10.24 years. Similar studies on COPD patients showed comparable results, such as; Couillard S, et al.^17^ reported the 51.5% males, 49.5% females with mean age of 71.4 ±10.3 years, Oshagbemi OA, et al.^18^ reports the 55.5% male, and 44.5% female with mean age of 68.4 ±10.8 years, and Bélanger M, et al.^19^ reported the 52.0% males, and 46.0% females with mean age of 68.9 ±9.4 years.

Current and previous studies show that older age is a major risk factor of COPD patients. Current research reported that most of the patients suffering from COPD are in age group of 61-70 years 49 (32.7%), followed by 71-80 years 37 (24.7%), 51-60 years 35 (23.3%), and 41-50 years 29 (19.3%). Similar results were reported by other researchers such as; Hasegawa K, et al.^14^ reported the higher prevalence of COPD in 70-79 years having 30.5% patients, followed by 60-69 years having 26.0% patients, ≥80 years having 21.5% patients, 50-59 years having 16.0% patients, and 40-49 years having 5.5% patients, and Oshagbemi OA, et al.^18^ reported the higher prevalence of COPD in 60-70 years having 62.3% patients, followed by 40-59 years having 21.1% patients and ≥ 80 years with 16.7% patients.

Two important factors that play vital role in the development of COPD are Smoking and Biomass Exposure. Current study reports the smoking history in 42.0% patients including Current smoking in 48 (32.0%) and Ex-Smoking in 15 (10.0%) patients, whereas Biomass Exposure in 76 (50.7%) patients. Different studies report the different prevalence of Smoking and Biomass Exposure, such as; Couillard S, et al.,^17^ Oshagbemi OA, et al.,^18^ Bélanger M, et al.,^19^ and Casanova C, et al.^20^ reported smoking in 53.3%, 44.4%, 54.3%, and 29.0% respectively, whereas Bakr RM, et al.^21^ reported the Biomass Exposure in 41.0% patients. All studies revealing the important role of smoking and biomass exposure in development of COPD.

According to GOLD standard COPD patients were categorized into; Mild 11 (7.3%), Moderate 68 (45.3%), Severe 59 (39.3%), and Very Severe 12 (8.0%). Similar results were reported by Couillard S, et al.^17^ who observed Mild 7.8%, Moderate 47.9%, Severe 35.3%, and Very Severe 9.0% COPD disease, and Bélanger M, et al.^19^ who observed Mild 6.3%, Moderate 45.1%, Severe 40.9%, and Very Severe 7.7% COPD among patients. All studies revealing the similar pattern of COPD severity.

Second important aspect of study was presence of Eosinophilic COPD in COPD patients. Current study reports the Eosinophilic COPD in 59 (39.3%) patients, and Non-Eosinophilic COPD in 91 (60.7%) patients. Similar higher prevalence was reported by Couillard S, et al.,^17^ and Bélanger M, et al.^19^ they observed 32.9%, and 36.1% COPD patients having blood eosinophilia respectively, whereas lower prevalence was reported by Negewo NA, et al.,^9^ and Hasegawa K, et al.,^14^ observed blood eosinophilia in 15.6%, and 17% COPD patients respectively. Prevalence of blood eosinophilia depends upon the severity of COPD, as severity increases level of blood eosinophilia also increases. Therefore, all studies with higher prevalence of COPD severity reports the higher prevalence of blood eosinophilia. Our study has documented the presence of high frequency of eosinophilia in COPD, this will have impact on awareness regarding management of COPD as it will require inhaled steroids.

### Limitations of the study

Our study has the limitation of being a single center study so its result cannot be generalized. This creates an opportunity to conduct multicenter studies in different regions of the country to determine an overall impact of this phenomenon.

## CONCLUSION

It was concluded that frequency of blood eosinophilia in newly diagnosed COPD patients was high, among which most of the patients were having moderate to severe COPD. Therefore, eosinophilia should be considered while managing COPD patients.

### Author’s Contribution

**GMK:** Wrote manuscript, did statistical analysis and he is responsible for integrity of research.

**FFZ:** Designed and conceived the study, did statistical analysis and final approval of manuscript.

**SBUZ & LG:** Collected data, literature search and did initial draft write-up.

## References

[1] Mirza S, Benzo R (2017). Chronic obstructive pulmonary disease phenotypes:implications for care. Mayo Clin Proc.

[2] Vestbo J, Agusti A, Wouters EF, Bakke P, Calverley PM, Celli B (2014). Should we view chronic obstructive pulmonary disease differently after ECLIPSE?A clinical perspective from the study team. Am J Respir Crit Care Med.

[3] (2019). Global initiative for chronic obstructive lung disease. GOLD 2019 Global strategy for the diagnosis, management, and prevention of chronic obstructive lung disease [Internet].

[4] World Health Organization (2017). Chronic obstructive pulmonary disease (COPD) [Fact sheet].

[5] Riley CM, Sciurba FC (2019). Diagnosis and outpatient management of chronic obstructive pulmonary disease:a review. JAMA.

[6] World Health Organization (2015). Chronic obstructive pulmonary disease (COPD).

[7] Leem AY, Park B, Kim YS, Jung JY, Won S (2018). Incidence and risk of chronic obstructive pulmonary disease in a Korean community-based cohort. Int J Chron Obstruct Pulmon Dis.

[8] Eltboli O, Brightling CE (2013). Eosinophils as diagnostic tools in chronic lung disease. Expert Rev Respir Med.

[9] Negewo NA, McDonald VM, Baines KJ, Wark PAB, Simpson JL, Jones PW (2016). Peripheral blood eosinophils:a surrogate marker for airway eosinophilia in stable COPD. Int J Chron Obstruct Pulmon Dis.

[10] Vedel-Krogh S, Nielsen SF, Lange P, Vestbo J, Nordestgaard BG (2016). Blood eosinophils and exacerbations in chronic obstructive pulmonary disease. The Copenhagen general population study. Am J Respir Crit Care Med.

[11] Kerkhof M, Sonnappa S, Postma DS, Brusselle G, Agusti A, Anzueto A (2017). Blood eosinophil count and exacerbation risk in patients with COPD. Eur Respir J.

[12] Oh YM, Lee KS, Hong Y, Hwang SC, Kim JY, Kim DK (2018). Blood eosinophil count as a prognostic biomarker in COPD. Int J Chron Obstruct Pulmon Dis.

[13] Eltboli O, Mistry V, Barker B, Brightling CE (2015). Relationship between blood and bronchial submucosal eosinophilia and reticular basement membrane thickening in chronic obstructive pulmonary disease. Respirol.

[14] Hasegawa K, Camargo CA (2016). Prevalence of blood eosinophilia in hospitalized patients with acute exacerbation of COPD. Respirol.

[15] Vestbo J, Hurd SS, Agusti AG, Jones PW, Vogelmeier C, Anzueto A (2013). Global strategy for the diagnosis, management, and prevention of chronic obstructive pulmonary disease:GOLD executive summary. Am J Respir Crit Care Med.

[16] Han MK, Agusti A, Calverley PM, Celli BR, Criner G, Curtis JL (2010). Chronic obstructive pulmonary disease phenotypes:the future of COPD. Am J Respir Crit Care Med.

[17] Couillard S, Larivee P, Courteau J, Vanasse A (2017). Eosinophils in COPD exacerbations are associated with increased readmissions. Chest.

[18] Oshagbemi OA, Burden AM, Braeken DCW, Henskens Y, Wouters EFM, Driessen JHM (2017). Stability of blood eosinophils in patients with chronic obstructive pulmonary disease and in control subjects, and the impact of sex, age, smoking, and baseline counts. Am J Respir Crit Care Med.

[19] Belanger M, Couillard S, Courteau J, Larivee P, Poder TG, Carrier N (2018). Eosinophil counts in first COPD hospitalizations:a comparison of health service utilization. Int J Chron Obstruct Pulmon Dis.

[20] Casanova C, Celli BR, de-Torres JP, Martinez-Gonzalez C, Cosio BG, Pinto-Plata V (2017). Prevalence of persistent blood eosinophilia:relation to outcomes in patients with COPD. Eur Respir J.

[21] Bakr RM, Elmahallawy II (2012). Prevalence characteristics of COPD in never smokers. Egypt J Chest Dis Tuberc.

